# Nomenclature updates resulting from the evolution of avian influenza A(H5) virus clades 2.1.3.2a, 2.2.1, and 2.3.4 during 2013–2014

**DOI:** 10.1111/irv.12324

**Published:** 2015-08-04

**Authors:** Gavin J D Smith, Ruben O Donis, World Health Organization/World Organisation for Animal Health/Food and Agriculture Organization (WHO/OIE/FAO) H5 Evolution Working Group

**Affiliations:** a Program of Emerging Infectious Diseases, Duke-NUS Graduate Medical SchoolSingapore city, Singapore; b Influenza Division, Centers for Disease Control and PreventionAtlanta, GA, USA

**Keywords:** clade nomenclature, H5 subtype, hemagglutinin, highly pathogenic avian influenza, molecular epidemiology, phylogenetics, viral evolution

## Abstract

**Aim:**

The A/goose/Guangdong/1/96-like hemagglutinin (HA) genes of highly pathogenic avian influenza (HPAI) A(H5) viruses have continued to rapidly evolve since the most recent update to the H5 clade nomenclature by the WHO/OIE/FAO H5N1 Evolution Working Group. New clades diverging beyond established boundaries need to be identified and designated accordingly.

**Method:**

Hemagglutinin sequences deposited in publicly accessible databases up to December 31, 2014, were analyzed by phylogenetic and average pairwise distance methods to identify new clades that merit nomenclature changes.

**Results:**

Three new clade designations were recommended based on division of clade 2·1·3·2a (Indonesia), 2·2·1 (Egypt), and 2·3·4 (widespread detection in Asia, Europe, and North America) that includes newly emergent HPAI virus subtypes H5N2, H5N3, H5N5, H5N6, and H5N8.

**Conclusion:**

Continued global surveillance for HPAI A(H5) viruses in all host species and timely reporting of sequence data will be critical to quickly identify new clades and assess their potential impact on human and animal health.

## Introduction

Highly pathogenic avian influenza (HPAI) A(H5N1) viruses continue to circulate in poultry and wild birds in parts of Asia, the Middle East, and Africa. The A(H5) hemagglutinin (HA) gene of these viruses, derived from the A/goose/Guangdong/1/96 (gs/GD/96) H5 HA lineage, has continued to rapidly evolve since the most recent update to the H5 clade nomenclature by the WHO/OIE/FAO H5N1 Evolution Working Group.^1^ The majority of circulating viruses detected since the beginning of 2013 shared the N1 neuraminidase (NA) gene and genotype V- or Z-like internal genes (or reassortants between them) from viruses that evolved in Asia since 1996.^2^–^4^ Full genome analysis of viruses collected before 2010 revealed limited propensity of the genotype V- or Z-like H5N1 viruses to generate persistent reassortant genotypes with divergent avian influenza genes.^5^ Recently, however, several HPAI A(H5) lineage viruses have acquired NA genes from unrelated avian influenza viruses via reassortment. A novel H5N5 reassortant subtype was detected in 2008,^6^,^7^ and H5N2, H5N3, H5N6, and H5N8 reassortant subtype viruses were reported in China during or after 2010.^8^–^13^ These viruses shared H5 genes originating from clade 2.3.4 H5N1 viruses that circulated in China and elsewhere since 2005. In 2014, H5N6 and/or H5N8 subtype viruses were detected in birds from 12 countries in Asia (China, Korea, Japan, and Russia: H5N8; China, Laos, and Vietnam: H5N6), Europe (Germany, Netherlands, UK, Italy: H5N8) and North America (Canada and United States of America: H5N2 and H5N8).^14^–^17^ In January and February 2015, HPAI H5N1 viruses were detected for the first time in the United States and Canada, respectively,^18^ although soon it became apparent that the N1 NA gene was of North American origin.^19^ Several recent independent reports describe the evolution of these emerging clade 2.3.4 HPAI viruses and identify divergent viruses using interim clade nomenclature conventions 15,20,21 pending definitive designations by the WHO/OIE/FAO H5 Evolution Working Group. This report describes the phylogenetic analysis of H5 HA sequence data available since the last nomenclature update (data deposited in databases through December 31, 2012) 1 and nomenclature for the newly emerged clades. Clade designations for newly emerged phylogenetic groups were recommended according to previously established criteria.^4^

## Materials and methods

A total of 4294 H5 HA sequences from GISAID and GenBank databases with virus sequence deposit dates up to and including December 31, 2014, were used in this nomenclature analysis. Since the last H5 clade nomenclature revision, 567 sequences were newly submitted and an additional 16 gs/GD/96-like H5 HPAI viruses with N2 and N5 NA subtypes were also included for the first time in the analysis. All new sequence names, their assigned clades, accession numbers, and data sources are provided in Supplementary Data S2–S4. Sequences were curated using custom Perl scripts and database filters as in the previous nomenclature updates whereby sequences were removed before further analysis if analysis detected signs of recombination,^16^ duplicates, more than five ambiguous nucleotides, less than 60% alignment length, and frame-shifts.

**Figure 1:**
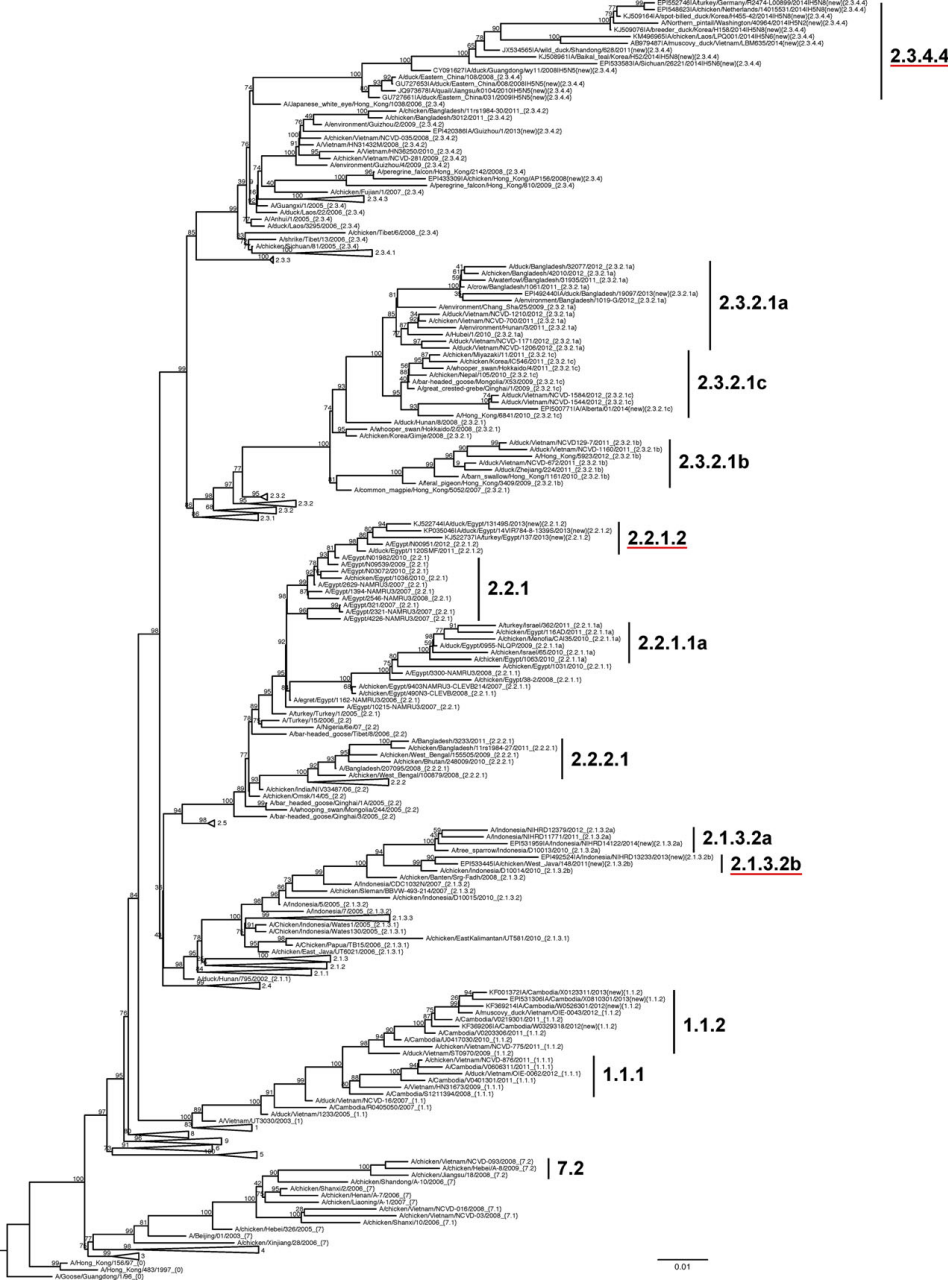
Phylogenetic relationships of recently diverged A/goose/Guangdong /1/1996 (Gs/GD/96)-like H5 hemagglutinin (HA) genes. A maximum likelihood tree of 242 HA nucleotide sequences from Gs/GD/96 lineage of HPAI H5 viruses was constructed with 10 000 local support bootstraps (above branches) using FastTree2 (GTR+GAMMA) and rooted to Gs/GD/96. Selected sequences are representative of all clades to render a condensed but accurate phylogenetic topology while including viruses from diverse countries, vaccine candidates, and human cases. All viruses are A(H5N1) unless labeled otherwise. Newly designated clades are underlined. Collapsed clades indicate viruses that have not been detected since 2012 or earlier. Scale bar denotes nucleotide substitutions per site. Sequence accession numbers are provided in Supplementary Data S2.

Data were aligned via mafft v7.187 22 and trimmed to the beginning of the mature H5 HA protein gene sequence using jalview v2.8.1.^23^ Approximate maximum likelihood trees (GTR+GAMMA with 10 000 local support bootstraps) were constructed using fasttree v2.1.4.^24^ Automated clade annotation of new sequences used label v0.4.4/H5v2013^25^ along with manual phylogenetic inspection. An H5 HA phylogeny visualization for a smaller dataset (Figure1), as well as pairwise p-distance matrices, was calculated in mega 5.1,^26^ and group averages were calculated with a custom Perl script. Figure1 is a tree of 242 representative H5 HA genes rooted to Gs/GD/96 (maximum likelihood, 10 000 local support bootstraps, GTR+GAMMA, FastTree2).

New clade designations required the presence of viruses sampled during 2013 and/or 2014, formation of monophyletic clusters with bootstrap values ≥60%, and within-clade average pairwise distances of ≤1·5%; however, this last upper limit is relaxed when phylogenetic and surveillance data are insufficient to support the split of the clade.^1^ As before, new clades were evaluated in the context of five major phylogenetic groups identified: (i) clades 0, 1, and 3 through 9; (ii) clades 2.1 and 2.4; (iii) clades 2.2 and 2.5; (iv) clade 2.3 with all higher order clades except 2.3.2.1; and (v) clade 2.3.2.1 (see Figure S1 A–E).^1^ As used for the previous update to the H5N1 nomenclature system,^1^ fifth-order groups are designated using an additional letter to the right of the fourth-order clade (i.e., 2.3.2.1a).

## Results

The reconstructed phylogenies revealed monophyletic groups in the majority of circulating clades. Groups containing new sequences were analyzed to determine the within-clade average pairwise nucleotide distances (APD; Figure S1A–E[Supplementary-material sd1]). Based on previously defined nomenclature criteria, we concluded that clades 2.1.3.2a, 2.2.1, and 2.3.4 required splitting (Table1). Clade 2.1.3.2a had an internal APD of 2·03% with viruses circulating in Indonesia from 2009 to 2014 and was split into clades 2.1.3.2a and 2.1.3.2b. Since the split of clade 2.2.1.1 from clade 2.2.1 in the 2012 nomenclature revision,^4^ the remaining viruses of clade 2.2.1 continued to evolve through 2013 and 2014 in Egypt and had achieved an internal APD of 1·70%. Therefore, a well-supported phylogenetic group of these clade 2.2.1 viruses was delimited as an additional clade, termed 2.2.1.2. Finally, clade 2.3.4 had a high within-clade APD at 2·95% due to the recent emergence and rapid geographic expansion of divergent H5 genes.^6^–^12^,^21^,^18^,^27^,^28^ This group of H5 viruses contained different NA subtypes including N1, N2, N3, N5, N6, and N8 and formed a distinct monophyletic lineage that was subdivided into a new clade designated 2.3.4.4.

**Table 1:** Genetic divergence within WHO/OIE/FAO H5 clades since January 1, 2013

Previous clades	Intra-clade (%)	Nearest clade	Inter-clade (%)	Updated clades	Intra-clade	Nearest clade	Inter-clade
0	2.05	3	2.29	2007 (last reported in)			
1	1.01	8	2.00	2010			
1.1	0.93	1.1.1	2.25	2008			
1.1.1	1.47	1.1	2.25	2012			
1.1.2	1.66	1.1.1	3.13	Limited detection in Mekong River Delta in 2014			
3	1.16	8	1.82	2001			
4	1.78	3	2.69	2006			
5	1.76	8	2.60	2009			
6	1.33	8	2.07	2004			
7	3.01	3	3.29	2008			
7.1	1.26	7.2	5.08	2008			
7.2	2.78	7.1	5.08	Limited detection in 2013 and 2014			
8	0.69	9	1.46	2002			
9	1.55	8	1.46	2009			
2.1.1	1.48	2.1.2	2.08	2007			
2.1.2	1.09	2.1.1	2.08	2006			
2.1.3	1.14	2.1.1	2.83	2007			
2.1.3.1	1.90	2.1.3.3	2.61	2011			
2.1.3.2	1.74	2.1.3.1	2.79	2010			
2.1.3.2a (Indonesia)	2.03	2.1.3.2	3.52	2.1.3.2a	1.38%	2.1.3.2b	2.72%
				2.1.3.2b	1.43%	2.1.3.2a	2.72%
2.1.3.3	1.60	2.1.3.1	2.61	2010			
2.2	1.21	2.2.2	2.18	2008			
2.2.1 (Egypt)	1.70	2.2	2.25	2.2.1	1.44%	2.2	1.93%
				2.2.1.2	1.05%	2.2.1	2.14%
2.2.1.1	1.53	2.2.1.1a	2.38	2010			
2.2.1.1a	1.29	2.2.1.1	2.38	2011			
2.2.2	1.01	2.2.2.1	2.14	2010			
2.2.2.1	1.45	2.2.2	2.14	2011			
2.3.1	1.07	2.3.2	2.29	2005			
2.3.2	1.20	2.3.1	2.29	2006			
2.3.2.1	1.55	2.3.2.1a	3.17	2011			
2.3.2.1a	1.93	2.3.2.1c	3.12	Limited detection in Bangladesh during 2014			
2.3.2.1b	1.67	2.3.2.1	3.43	Limited detection in China and Hong Kong SAR in 2013			
2.3.2.1c	1.75	2.3.2.1a	3.12	Cambodia, China, Laos, Indonesia, Vietnam in 2014			
2.3.3	0.76	2.3.1	2.79	2006			
2.3.4 (widespread)	2.95	2.3.4.3	2.77	2.3.4	1.59%	2.3.4.3	2.05%
				2.3.4.4	2.63%	2.3.4	6.65%
2.3.4.1	1.09	2.3.4.3	3.80	2010			
2.3.4.2	1.66	2.3.4.3	2.78	Outliers detected in China in 2013*			
2.3.4.3	0.78	2.3.4	2.75	2009			
2.4	1.03	2.1.1	2.19	2005			
2.5	1.09	2.2	2.61	2006			

* Outliers represented by A/Guizhou/1/2013 and A/Guizhou/2/2013; viruses in this cluster were not reported in 2014.

The new clade 2.3.4.4 had an internal APD of 2·63%, reflecting the rapid evolution of this mixed-subtype group. However, because of the sampling biases reflected by large numbers of sequences derived from individual outbreaks and insufficient data available from regions with very recent introductions, further splits were postponed until more genetic sequence data become available to understand the extent of sustained circulation of specific clusters and support additional clade designations. The viruses remaining in clade 2.3.4 after the 2.3.4.4 split had an internal APD of 1·59%, slightly exceeding the 1·5% threshold; however, these isolates were not detected since 2010 and therefore did not meet the criteria for a new clade designation. Clades 1.1.2, 2.3.2.1a, b, c, and 7·2 were above the 1·5% threshold but were not split due to lack of sufficient circulation in 2013 and 2014 (clades 1.1.2 and 7.2), formation of new monophyletic groups during this time period (clade 2.3.2.1a and 2.3.2.1b), or major changes in the enzootic foci of poultry outbreaks (a single human case of clade 2.3.2.1c in Canada and detection in smuggled birds in Austria were considered isolated importation events). No changes were identified in clades that were previously considered “extinct” as no new data were identified. In addition, no new virus sequences were reported in either 2013 or 2014 in clades 1.1.1, 7.1, 2.1.3.1, 2.1.3.3, 2.2.1.1, 2.2.1.1a, 2.2.2, 2.2.2.1, 2.3.2.1, 2.3.4.1, and 2.3.4.3 (Figure1, Table1).

## Discussion

Enzootic HPAI A(H5N1) viruses continue to cause outbreaks, predominantly in poultry, and were reported in over 11 countries including Bangladesh, Cambodia, China, Egypt, India, Indonesia, Laos, and Vietnam during 2014.^29^,^30^ Since the initial spread of H5N1 viruses throughout Eurasia and into North Africa, the viruses became entrenched in poultry populations in geographically isolated areas causing sporadic outbreaks and human infections.^5^ However, since 2013, there has been a resurgence of HPAI H5 virus activity, particularly related to the emergence of reassortant HPAI A(H5N2, H5N5, H5N6, and H5N8) viruses that have been detected across Asia and Europe and more recently in North America.^18^,^31^

The previous update of the WHO/OIE/FAO H5N1 Evolution Working Group analyzed data available up to December 31, 2012, and identified 11 H5 clades actively circulating during 2011 and 2012.^1^ This current nomenclature proposes three new clades, two that appear geographically restricted: clade 2.1.3.2b in Java, Indonesia, and clade 2.2.1.2 in Egypt. The diversification of endemic viruses within a given area is well recorded, and the observed diversification of these H5N1 virus populations is well within expectations. The third, clade 2.3.4.4, is widespread and composed of a variety of H5 viruses with different NA subtypes that include N1, N2, N3, N5, N6, and N8. The spread of H5N1 viruses to Europe in 2005–2006 and subsequent years following wild bird outbreaks in Qinghai Lake, China, has been extensively documented. The recent detection of HPAI A(H5) gs/GD/96 lineage viruses in wild ducks in Canada and the western seaboard of the USA is not unprecedented considering previous evidence of gene flow between the Eurasian and American low pathogenicity avian influenza gene pools and overlapping migratory bird flyways.^32^ H5N8 and H5N2 viruses have also recently been detected in poultry, and H5N2 viruses have been identified in wild birds from the central and Mississippi flyways of North America.^15^,^33^,^34^ It remains to be seen whether these viruses become established in North America or what affect they may have on the influenza virus gene pool of aquatic bird populations worldwide.

In articles published between updated nomenclature releases, emerging clades such as 2.3.4.4 have provisionally been given names that may differ from future nomenclature revisions.^6^,^8^,^9^,^11^,^12^,^21^,^27^,^28^ While the WHO/FAO/OIE H5N1 Evolution Working Group considers historical convention in its nomenclature revisions, authors are encouraged to add the word *provisional* to describe emerging clades in order to facilitate continuity within the literature in the event that such names are not later adopted by the community at large. Continued global surveillance, monitoring, and characterization of HPAI A(H5) viruses from poultry and wild birds, as well as those from sporadic human infections, will be critical to assess the prevalence and public health significance of these new clades in the future.

## Supporting Information

**Figure S1.** (A–E) Phylogenetic relationships of recently diverged A/goose/Guangdong /1/1996 (Gs/GD/96)-like H5 hemagglutinin (HA) genes. Five maximum likelihood trees containing a total of 4292 HA nucleotide sequences from (Gs/GD/96) lineage HPAI H5 viruses were constructed with 10 000 local support bootstraps (above branches) using fasttree2 (GTR+GAMMA) and rooted to Gs/GD/96. Newly designated clades are annotated and shown in color with the within-clade average pairwise nucleotide distances. Scale bar denotes nucleotide substitutions per site. A (clade 0, 1, 3–9); B (2.1.3); C (2.2.1); D (2.3.2.1); E (2.3.4).

**Data S1.** Members of the World Health Organization/World Organisation for Animal Health/Food and Agriculture Organization (WHO/OIE/FAO) H5N1 Evolution Working Group.

**Data S2.** List of viruses, clade designation, gene accession numbers, and database sources for sequences used in Figure 1.

**Data S3.** List of new sequence data since the previous clade nomenclature update,^1^ including viruses, clade designation, gene accession numbers, and database sources for sequences used in the phylogenetic analysis.

**Data S4.** List of authors, originating and submitting laboratories of the sequences from GISAID’s EpiFlu™ Database used in this report.
