# Supplementary figures and images for: Nomenclature updates resulting from the evolution of avian influenza A(H5) virus clades 2.1.3.2a, 2.2.1, and 2.3.4 during 2013–2014
Source: Influenza Other Respir Viruses. 2015 Aug 4;9(5):271–6. doi: 10.1111/irv.12324 (PMC4548997; doi:10.1111/irv.12324)

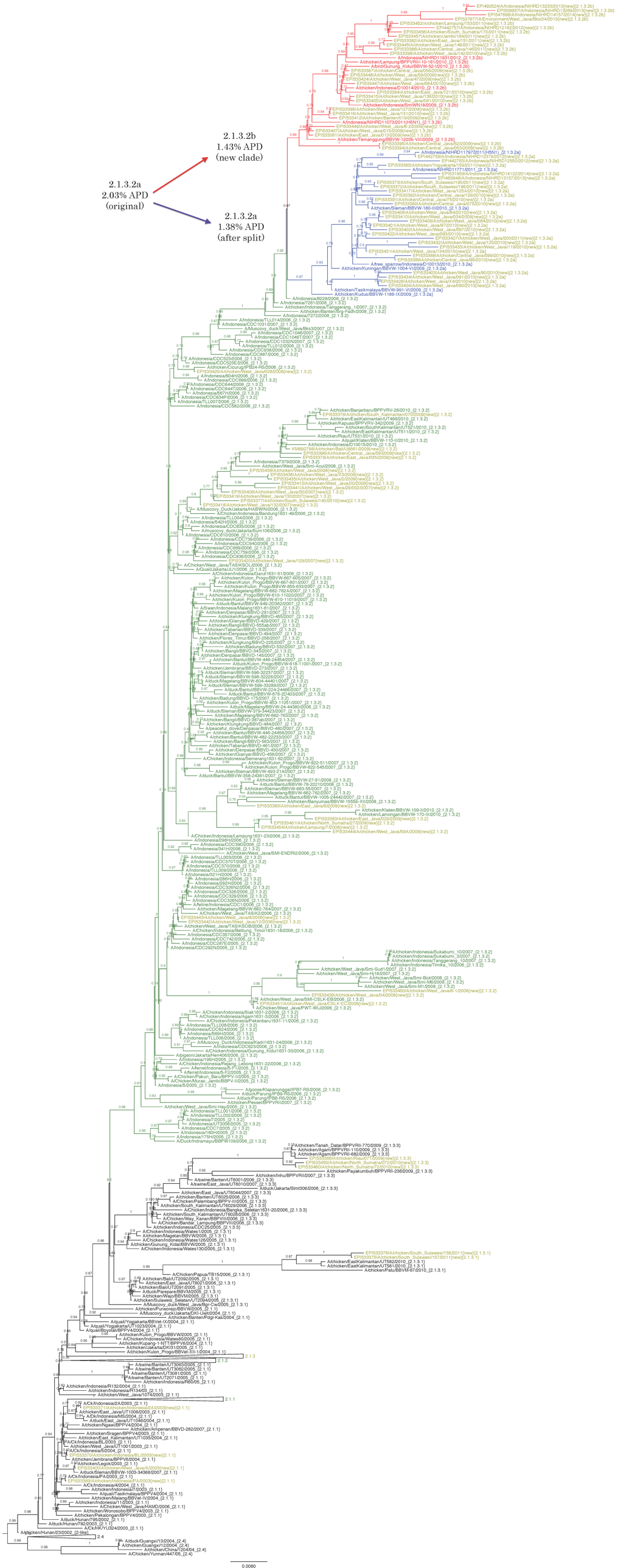

Supplement: Supplementary file 1 [file irv0009-0271-sd1.zip › irv12324-sup-0002-FigS1B.tif]

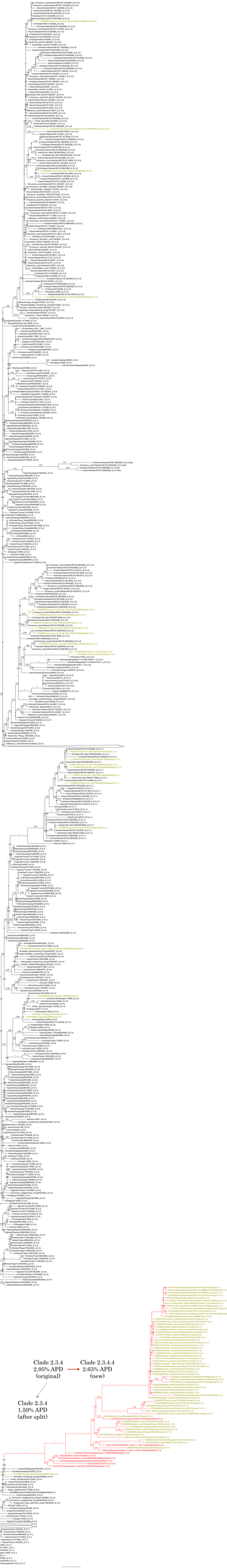

Supplement: Supplementary file 6 [file irv0009-0271-sd6.pdf]
