# Supplementary material for: Nomenclature updates resulting from the evolution of avian influenza A(H5) virus clades 2.1.3.2a, 2.2.1, and 2.3.4 during 2013–2014
Source: Influenza Other Respir Viruses. 2015 Aug 4;9(5):271–6. doi: 10.1111/irv.12324 (PMC4548997; doi:10.1111/irv.12324)
Supplement: Supplementary file 2 [file irv0009-0271-sd2.docx]

Nomenclature updates resulting from the evolution of avian influenza A(H5) virus clades 2.1.3.2a, 2.2.1, and 2.3.4 during 2013-2014

WHO/OIE/FAO H5 Evolution Working Group

**SUPPLEMENTARY DATA S1**

**H5 Evolution Working Group Members and Collaborators**

The working group was established in 2008 by request of the World Health Organization’s Global Influenza Programme, Department of Epidemic and Pandemic Alert and Response (WHO, GIP, EPR), the World Organization for Animal Health (OIE), and the Food and Agriculture Organization (FAO). It currently consists of the following persons:

1. Justin Bahl, Center for Infectious Diseases, The University of Texas School of Public Health, Houston, Texas, USA and Duke-NUS Graduate Medical School; Singapore;
2. Terry Besselaar, WHO, GISRS, Geneva, Switzerland;
3. Ian H. Brown, , Animal and Plant Health Agency , Addlestone, United Kingdom;
4. Giovanni Cattoli, Istituto Zooprofilattico Sperimentale delle Venezie, Padova, Italy;
5. Hualan Chen, Harbin Veterinary Research Institute, CAAS, China;
6. Nancy J. Cox, WHO Collaborating Centre for the Surveillance, Epidemiology and Control of Influenza, Centers for Disease Control and Prevention, Atlanta, Georgia, USA;
7. Filip Claes, FAO, Rome, Italy;
8. Gwenaelle Dauphin, FAO, AGAH, Rome, Italy;
9. C. Todd Davis, Centers for Disease Control and Prevention, Atlanta, Georgia, USA;
10. Ruben O. Donis, Centers for Disease Control and Prevention, Atlanta, Georgia, USA;
11. Ron A.M. Fouchier, Erasmus University, Netherlands;
12. Yi Guan, The University of Hong Kong, HK SAR, China;
13. Keith Hamilton, OFFLU, OIE, Paris, France;
14. Yunho Jang, Centers for Disease Control and Prevention, Atlanta, Georgia, USA;
15. Yoshihiro Kawaoka, University of Wisconsin, Madison, Wisconsin, USA and Institute of Medical Science, University of Tokyo, Tokyo, Japan;
16. Anne Kelso, WHO Collaborating Centre for Reference and Research on Influenza, Melbourne, Australia;
17. Mia Kim-Torchetti, National Veterinary Services Laboratories, Ames, Iowa, USA
18. Tommy Lam, The University of Hong Kong, HK SAR, China;
19. John McCauley, WHO Collaborating Centre for Reference and Research on Influenza,
    MRC National Institute for Medical Research, London, UK;
20. Wenqing Zhang, WHO, GISRS, Geneva, Switzerland;
21. Teguh Prajitno, Japfa, Jakarta, Indonesia;
22. Colin A. Russell, Department of Zoology, University of Cambridge, England, United Kingdom;
23. Derek Smith, Department of Zoology, University of Cambridge, England, United Kingdom;
24. Gavin J.D. Smith, Duke-NUS Graduate Medical School; Singapore;
25. Yuelong Shu, Chinese Center for Disease Control and Prevention, Beijing, China;
26. Takato Odagiri, WHO Collaborating Centre for Reference and Research on Influenza,
    National Institute of Infectious Diseases, Tokyo, Japan;
27. Stacey Schultz, WHO Collaborating Center for Studies on the Ecology of Influenza in Animals, St. Jude Children’s Research Hospital, Memphis, USA;
28. Samuel Shepard, Centers for Disease Control and Prevention, Atlanta, Georgia, USA;
29. David Suarez, Southeast Poultry Laboratory, USDA, Athens, Georgia, USA;
30. Dhanasekaran Vijaykrishna, Duke-NUS Graduate Medical School; Singapore;
31. Dayan Wang, Chinese Center for Disease Control and Prevention, Beijing, China;
32. Richard Webby, WHO Collaborating Center for Studies on the Ecology of Influenza in Animals, St. Jude Children’s Research Hospital, Memphis, USA;
33. Robert Webster, St. Jude Children's Research Hospital, Memphis, USA;
34. Frank Wong, Australian Animal Health Laboratory, Geelong, Australia;
35. Maria Huachen Zhu, The University of Hong Kong, HK SAR, China.
